# Taurine Promotes Neurite Outgrowth and Synapse Development of Both Vertebrate and Invertebrate Central Neurons

**DOI:** 10.3389/fnsyn.2020.00029

**Published:** 2020-07-22

**Authors:** Brittany Mersman, Wali Zaidi, Naweed I. Syed, Fenglian Xu

**Affiliations:** ^1^Department of Biology, College of Arts and Sciences, Saint Louis University, St. Louis, MO, United States; ^2^Henry and Amelia Nasrallah Center for Neuroscience, Saint Louis University, St. Louis, MO, United States; ^3^Department of Cell Biology and Anatomy, Hotchkiss Brain Institute and Alberta Children’s Hospital Research Institute, University of Calgary, Calgary, AB, Canada

**Keywords:** taurine, neural development, synapse, vertebrate, invertebrate, molluska, synaptic transmission and plasticity

## Abstract

Taurine is a sulfur-containing amino acid that is widely expressed throughout the human brain, heart, retina, and muscle tissues. Taurine deficiency is associated with cardiomyopathy, renal dysfunction, abnormalities of the developing nervous system, and epilepsy which suggests a role specific to excitable tissues. Like vertebrates, invertebrates maintain high levels of taurine during embryonic and larval development, which decline during aging, indicating a potential developmental role. Notwithstanding its extensive presence throughout, taurine’s precise role/s during early brain development, function, and repair remains largely unknown in both vertebrate and invertebrate. Here, we investigated whether taurine affects neurite outgrowth, synapse formation, and synaptic transmission between postnatal day 0 rat cortical neurons *in vitro*, whereas its synaptogenic role was tested more directly using the *Lymnaea* soma-soma synapse model. We provide direct evidence that when applied at physiological concentrations, taurine exerts a significant neurotrophic effect on neuritic outgrowth and thickness of neurites as well as the expression of synaptic puncta as revealed by immunostaining of presynaptic synaptophysin and postsynaptic PSD95 proteins in rat cortical neurons, indicating direct involvement in synapse development. To demonstrate taurine’s direct effects on neurons in the absence of glia and other confounding factors, we next exploited individually identified pre- and postsynaptic neurons from the mollusk *Lymnaea stagnalis*. We found that taurine increased both the incidence of synapse formation (percent of cells that form synapses) and the efficacy of synaptic transmission between the paired neurons. This effect was comparable, but not additive, to *Lymnaea* trophic factor-induced synaptogenesis. This study thus provides direct morphological and functional evidence that taurine plays an important role in neurite outgrowth, synaptogenesis, and synaptic transmission during the early stages of brain development and that this role is conserved across both vertebrate and invertebrate species.

## Introduction

Taurine, 2-aminoethanesulfonic acid, is an abundant, free amino acid in human and most animal brains (Huxtable, 1989) and is also present in the heart, retina, and muscle tissues (Ripps and Shen, 2012). Humans obtain taurine either from the diet or from biochemical synthesis (Jacobsen and Smith, 1968). Its biosynthesis is derived from cysteine in which cysteine dioxygenase and cysteinesulfinic acid decarboxylase form hypotaurine. Hypotaurine is then converted by hypotaurine dehydrogenase to form taurine (Vitvitsky et al., 2011). As a structural analog of the inhibitory neurotransmitter γ-aminobutyric acid (GABA), taurine mimics GABA action by activating GABA_A_ receptors (El Idrissi and Trenkner, 2004; Jia et al., 2008), and transport of taurine into neurons occurs *via* Slc6a6/TauT, the same family of proteins that contribute to GABA transport (Smith et al., 1992; Uchida et al., 1992; Tomi et al., 2008). Taurine has been shown to play a role in many physiological processes including osmoregulation (Solis et al., 1988), membrane excitability changes (Galarreta et al., 1996), and neuronal development where it acts as a putative neurotrophic factor (Chen et al., 1998; Rak et al., 2014). Taurine is also neuroprotective, as it regulates calcium homeostasis (Chen et al., 2001), acts as an antioxidant (Martincigh et al., 1998), and functions as a modulator of inflammation (Marcinkiewicz and Kontny, 2014). Some of the neuroprotective pathways by which taurine acts have also been identified. For example, taurine activates the PI3-K/Akt pathway to increase cell survival during oxidative stress (Das et al., 2011) and downregulates Bax (pro-apoptotic Bcl-2-associated protein) and caspase-3 in traumatic brain injury (Niu et al., 2018).

As one of the most abundant organic molecules in the central nervous system (CNS), taurine has been deemed a key regulator of neurodevelopment for decades. As Sturman et al. (1985) showed in their seminal article, taurine-deficient kittens displayed developmental abnormalities, indicating its vital role in proper CNS development. In the developing nervous system, taurine acts as a trophic factor (Sturman, 1993; Chen et al., 1998; Rak et al., 2014) and is present at a concentration three times higher in the immature brain than the adult nervous system; some studies suggest a concentration up to the 1–9 mM range (Benitez-Diaz et al., 2003; Albrecht and Schousboe, 2005; Furukawa et al., 2014). The addition of taurine increases stem cell proliferation in the developing mouse hippocampus as well as expression of synaptic proteins in rat hippocampal primary culture, presumably contributing to the overall connectome of the mouse brain (Shivaraj et al., 2012). These studies, in addition to the fact that taurine concentration in the brain decreases with age, suggest that taurine plays a major role in the proper development of neurons and networks (Banay-Schwartz et al., 1989). However, it remains unknown if taurine acts to regulate multiple developing steps including neural morphogenesis, synaptogenesis, synaptic transmission, and plasticity and if taurine’s effects in developing brains are species-specific (e.g., mouse vs. rat or vertebrate vs. invertebrate) or brain region-specific (e.g., hippocampus vs. cortex).

Like vertebrates, taurine is present in the nervous system of many invertebrates (Allen and Garrett, 1971; McCaman and Stetzler, 1977). Specifically, in marine invertebrates, taurine’s robust role in osmoregulation is well characterized (Lange, 1963; Allen and Garrett, 1971; Gilles, 1972; Smith and Pierce, 1987; Miles et al., 2018). Other potential roles of taurine in invertebrates have been suggested such as an H_2_S scavenger in hydrothermal vent invertebrates (Koito et al., 2018), a source of energy for marine prokaryotes (Clifford et al., 2019), and a regulator of temperature tolerance in the fish Preccottus glehnii (Karanova, 2009). Similar to vertebrates, taurine has been found in high concentrations in some invertebrate larvae species during development and metamorphosis (Welborn and Manahan, 1995). However, little research is carried to explore taurine’s developmental and physiological roles in the nervous system of invertebrates.

Considering the above evidence, we first asked the question: Does taurine morphologically affect the development of molecular components such as the cytoskeletal and synaptic proteins in vertebrate neurons? To answer this question, we studied the effects of taurine on primary neurons derived from postnatal rat cortex, a brain region and species severely understudied in this context. Our results indicate that taurine increases the number of neuritic branches and the thickness of neurites. Taurine also significantly regulates the expression and/or puncta localization of presynaptic/postsynaptic markers in developing neurons, with a more robust effect on the presynaptic markers. We next asked the questions: Does taurine affect synapse formation, synaptic transmission, and plasticity between central neurons? Does it affect the pre- or postsynaptic machinery? Because direct cell-cell interactions between defined sets of pre- and postsynaptic neurons cannot be studied in vertebrates in the absence of glial and other confounding factors such as GABA and their receptors, we exploited the invertebrate *Lymnaea stagnalis* model where this could easily be achieved using soma-soma synapse culture and electrophysiological methods. Using this model, we, for the first time, demonstrate that taurine promotes synapse formation and synaptic transmission in invertebrate neurons of *Lymnaea stagnalis* brains, and taurine’s actions do not involve the modulation of postsynaptic machinery, indicating a presynaptic origin. This study, together with previous knowledge about taurine’s effects on hippocampal neurons, clearly demonstrates that taurine is a critical neuro-morphogenic and synaptogenic factor in different brain regions across both vertebrate and invertebrate species, and taurine-mediated effects are cell- or synaptic site-specific.

## Materials and Methods

### Animals and Cell Culture

All animal procedures followed the standards established by the National Institute of Health Animal Use Guidelines in Canada and the US and have been approved by the Institutional Animal Care and Use Policy at the University of Calgary and Saint Louis University.

#### Rat Cortical Neuronal Cell Culture

The culture of cortical neurons was made using Sprague-Dawley rat pups on the day they were born (postnatal day 0, P0). Rat frontal cortices were removed and enzymatically dissociated with papain (50 μg/ml). To create a single-cell suspension, glass pipettes of decreasing size were used to triturate. Dissociated cortical neurons were then diluted in culture media and plated at an appropriate density onto culture dishes with glass coverslips coated with poly-D-lysine (30 μg/ml) and laminin (2μg/ml). After cells settled for 30 mins, 2 ml of culture medium was added to each culture dish. The culture medium included neurobasal medium, 2% B27, L-Glutamine (200 mM), 4% FBS, and penicillin-streptomycin (Invitrogen). Cortical neurons were kept in culture medium and maintained at 37°C in an airtight modular incubator chamber (Billups-Rothenberg) circulated with medical air and 5% carbon dioxide. Fifty percent of the culture medium was removed and replaced every 3–4 days. Control cells for each experiment were incubated in the same environment (37°C, 5% CO_2_) as treatment cells.

#### *Lymnaea* Ganglion Dissection and Cell Culture

*L. stagnalis* were kept at room temperature in a well-aerated aquarium filled with filtered pond water at 20–22°C on a 12-h light/dark regimen and were fed romaine lettuce. For cell culture experiments, ~2–3-month-old *L. stagnalis* were used while ~4–6-month-old animals were used to make brain-conditioned medium (CM, containing trophic factors). Details of *Lymnaea* cell culture procedures and CM preparations are described in previous publications (Syed et al., 1990; Ridgway et al., 1991; Xu et al., 2009). In brief, deshelled snails were anesthetized for 10 mins in Listerine solution (ethanol, 21.9%; and methanol, 0.042%) diluted to 10% in normal saline (NaCl 51.3 mM, KCl 1.7 mM, CaCl_2_ 4.0 mM, MgCl_2_ 1.5 mM, and HEPES 10 mM), adjusted to pH 7.9. To make trophic factor-containing CM, the central ring ganglia (Figure 5A) were incubated in defined medium (DM; L-15; Invitrogen; special order) containing NaCl 40 mM, KCl 1.7 mM, CaCl_2_ 4.1 mM, MgCl_2_ 1.5 mM, and HEPES 10.0 mM (12 ganglia/6.5 ml DM) for at least 3 days before removing ganglia and collecting CM. To culture *L. stagnalis* neurons, the central ring ganglia were first treated for 21 mins with the proteolytic enzyme trypsin (2 mg/ml) dissolved in DM. The central ring ganglia were then incubated for 15 mins in a DM solution containing trypsin inhibitor (2 mg/ml) and subsequently pinned on a dissection dish containing high osmolarity DM (D-glucose, 20 mM). A dissection microscope was used to visualize *L. stagnalis* brains, and fine forceps were used to remove a thin layer of the sheath surrounding the ganglia. With gentle suction through a Sigmacote-treated, fire-polished pipette attached to a microsyringe filled with high osmolarity DM, individual cells were removed from the ganglia. Once isolated, well-defined pre- and postsynaptic cells were juxtaposed in a soma-soma configuration and plated on poly-_L_-lysine coated culture dishes containing medium as described previously (Meems et al., 2003). In brief, somas of isolated cells were manually severed from their axons *via* a conventional intracellular electrode attached to a micromanipulator. Two isolated somata were then juxtaposed and cultured overnight in the absence or presence of taurine or CM, depending on the experiment. The cells used in this study are the visceral dorsal 4 (VD4, presynaptic) and the left pedal dorsal 1 (LPeD1, postsynaptic) neurons (Figure 5B). VD4 neurons contain neurotransmitter acetylcholine (ACh), and LPeD1 neurons express nicotinic ACh receptors (nAChR). VD4 and LPeD1 neurons *in vivo* form cholinergic synapses that control cardiorespiratory behavior in *L. stagnalis* (Buckett et al., 1990).

**Figure 1:**
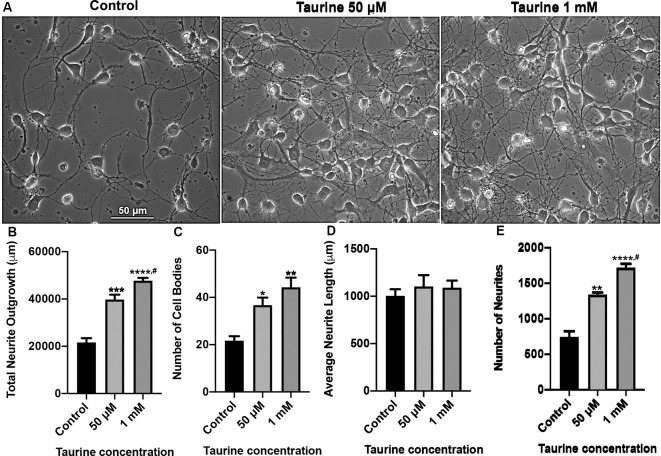
Taurine exposure promotes rat cortical neuronal growth and increases the number of neurites and cells in culture. Rat cortical neurons were cultured either in the absence (control) or presence of taurine at different concentrations. **(A)** Phase-contrast images were taken on day 3 in culture. **(B)** Taurine treatment significantly increased the total neurite outgrowth in phase-contrast images. The total neurite outgrowth for control was 21,634.2 ± 1788.7 μm, for 50 μM taurine was 39,775.7 ± 2,056.3 μm, and for 1 mM taurine was 47,714.8 ± 1,222.5 μm (*p* = 0.0007 for control vs. 50 μM taurine, *p* = 0.00009 for control vs. 1 mM taurine, and *p* = 0.04 for 50 μM vs. 1 mM taurine). **(C)** Taurine treatment also significantly increased the number of cell bodies per image compared to controls. The cell body count was 21.7 ± 1.9 for control, 36.7 ± 3.2 for 50 μM taurine, and 44.3 ± 4.1 for 1 mM taurine (*p* = 0.03 for control vs. 50 μM taurine, and *p* = 0.005 for control vs. 1 mM taurine). **(D)** However, calculating the average neurite length revealed a nonsignificant difference between control and taurine treatments. Average neurite length for control was 1,004.7 ± 69.3 μm, for 50 μM taurine was 1,102.7 ± 120.1 μm, and for 1 mM taurine was 1,090 ± 76.1 μm (*p* = 0.724). **(E)** The number of neurites was significantly increased when cortical neurons were cultured with taurine at both concentrations. Number of neurites in control was 740 ± 84.9, 50 μM taurine was 1,337 ± 34, and 1 mM taurine was 1,718.3 ± 55.6 (*p* = 0.001 for control vs. 50 μM taurine, *p* = 0.00007 for control vs. 1 mM taurine, and *p* = 0.01 for 50 μM vs. 1 mM taurine; *n* = 3 images per treatment; the statistical test was one-way ANOVA in all cases). *Indicates a significance level of *p* < 0.05 vs. control; **p < 0.01, ***p < 0.001, ****p < 0.0001, and ^#^indicates a significance level of *p* < 0.05 vs. 50 μM taurine.

**Figure 2:**
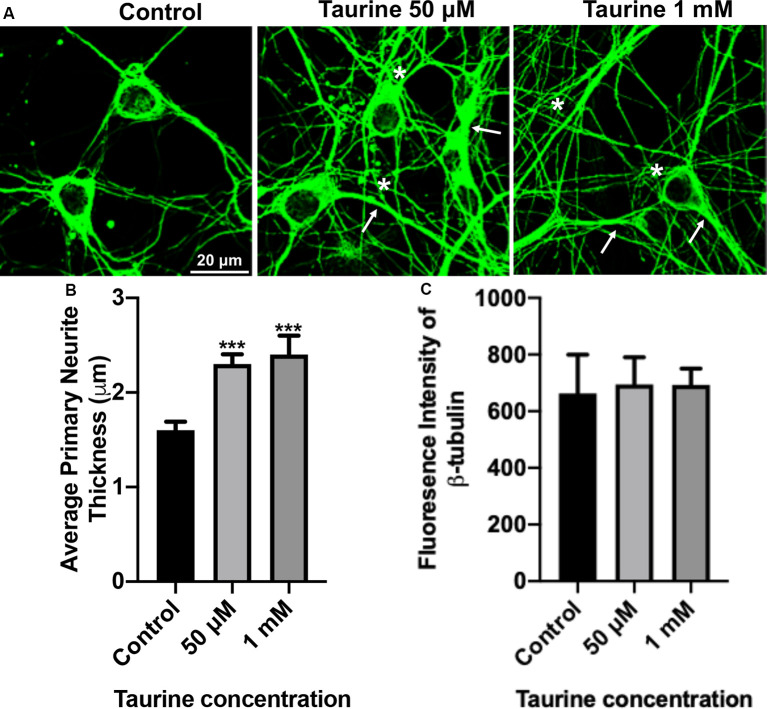
β-tubulin-labeled cortical neurons reveal an increase in the thickness of neurites after taurine treatment. After 3 days in taurine-rich (50 μM or 1 mM) or -free media, cortical neurons were stained with the cytoskeletal marker β-tubulin and imaged. **(A)** Representative images demonstrate the extensive outgrowth of cultures in all treatments and control. Arrows indicate examples of extensive neuritic processes while asterisks show robust interconnections. **(B)** Taurine at both 50 μM and 1 mM concentrations significantly increased the average thickness of primary neurites compared to control. The average neurite thickness for control was 1.6 ± 0.09 μm, for 50 μM taurine was 2.3 ± 0.1 μm, and for 1 mM taurine was 2.4 ± 0.2 μm (*p* = 0.0004 for control vs. 50 μM taurine, and *p* = 0.0002 for control vs. 1 mM taurine). **(C)** The intensity of β-tubulin was not significantly changed by any concentration of taurine. Specifically, the β-tubulin intensity of control was 663.3 ± 136.2, for 50 μM taurine was 694.3 ± 96.3, and for 1 mM taurine was 692.5 ± 58.3 (*p* = 0.971; *n* = 4 images per treatment and number of neurites analyzed was 40, 39, and 30 for control, 50 μM taurine, and 1 mM taurine, respectively; the statistical test was one-way ANOVA in all cases). ***Indicates a significance level of *p* < 0.001 vs. control.

**Figure 3:**
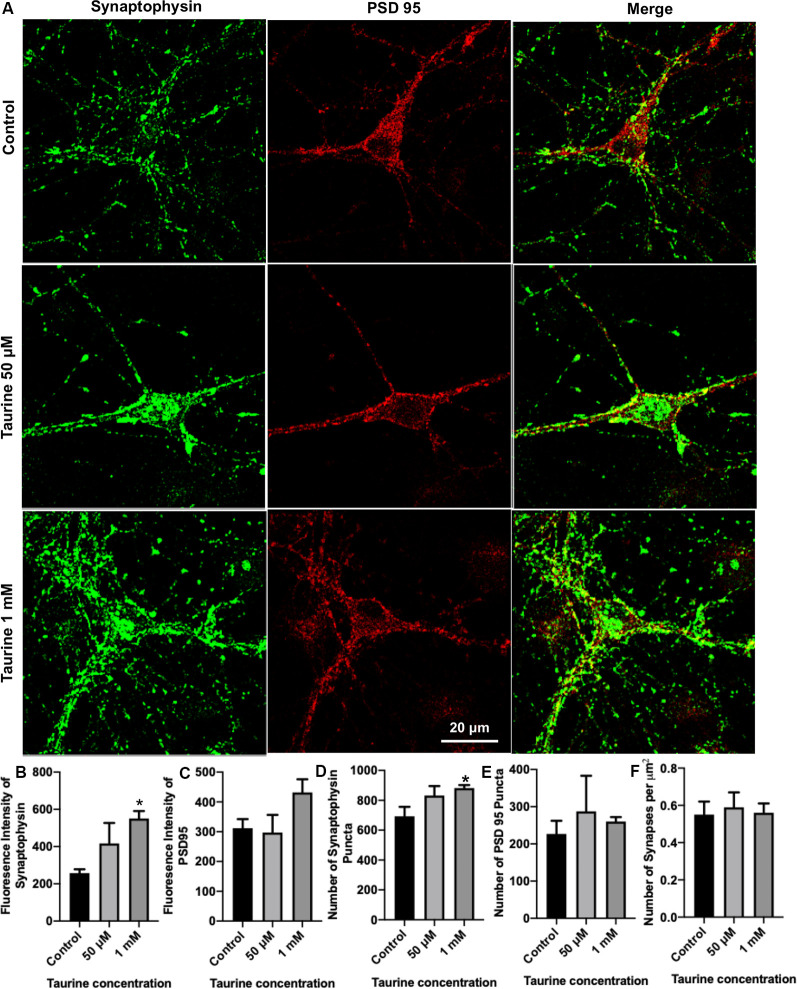
Taurine enhances the expression of presynaptic protein and presynaptic puncta in rat cortical neurons. Cortical neurons were cultured with 50 μM taurine, 1 mM taurine, or without taurine (control) for 10 days to allow for the development of mature networks. On day 10, cells were fixed and stained with antibodies against the presynaptic vesicle protein synaptophysin and the postsynaptic receptor density protein PSD95. Images were acquired using confocal microscopy and whole networks (entire fluorescent images) were analyzed for synaptic protein intensity, puncta, and colocalization. **(A)** Representative immunofluorescent images show the staining of synaptic proteins of control, 50 μM, 1 mM taurine-treated cortical neurons. **(B,C)** The addition of 1 mM taurine to culture media significantly increased the intensity of synaptophysin expression while not significantly altering PSD95 intensity. Specifically, the fluorescent intensity of synaptophysin in control was 257.6 ± 20.7, in taurine at 50 μM was 417.3 ± 110, and in taurine at 1 mM was 551.1 ± 39.6 (*p* = 0.02 for control vs. 1 mM taurine). The fluorescence intensity of PSD95 in control was 311.9 ± 30.5, in taurine at 50 μM was 297.1 ± 59.5, and in taurine at 1 mM was 431.9 ± 44.3 (*p* = 0.108). **(D,E)** The number of presynaptic synaptophysin and postsynaptic PSD95 puncta in a fluorescent image was measured by the ImageJ plugin SynQuant. The only significant change in puncta number occurred after 1 mM taurine treatment for synaptophysin puncta. Specifically, the number of synaptophysin puncta in control was 692.8 ± 63.3, in taurine at 50 μM was 832.3 ± 63.3, and in taurine at 1 mM was 881.7 ± 20.7 (*p* = 0.03 for control vs. 1 mM taurine). The number of PSD95 puncta in control was 226.8 ± 35.3, in taurine at 50 uM was 287.5 ± 95.6, and in taurine at 1 mM was 260.2 ± 11.8 (*p* = 0.743). **(F)** SynapCountJ, an ImageJ plugin, was utilized to determine synapses (per μm^2^) *via* colocalization of synaptophysin and PSD95 in traced neurites. There was no significant difference in the number of synapses between any treatments. Control cell number of synapses (per μm^2^) was 0.55 ± 0.07, 50 μM taurine was 0.59 ± 0.08, and 1 mM taurine was 0.56 ± 0.05 (*p* = 0.904; *n* = 4 images for control and 50 μM taurine-treated neurons; *n* = 6 images for 1 mM taurine-treated neurons; the statistical test was one-way ANOVA in all cases). *Indicates a significance level of *p* < 0.05 vs. control.

**Figure 4:**
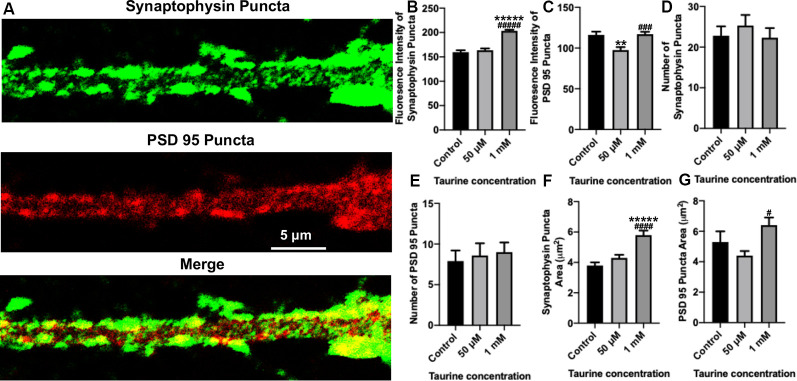
In neurites, a higher concentration of taurine (1 mM) increases presynaptic puncta intensity and area while keeping postsynaptic puncta unaffected. Puncta parameters in neurites were examined by zooming in on a 1,000 μm^2^ primary neurite extending from a pyramidal neuron and using the ImageJ plugin SynQuant. **(A)** Representative fluorescent images show an example of the 1,000 μm^2^, zoomed-in neurite from a 50 μM taurine-treated neuron. **(B)** The intensity of synaptophysin puncta in the cropped, neurite image was measured and showed a significant increase in 1 mM taurine-treated neurons. The intensities of synaptophysin puncta in control was 159.7 ± 4.0, 50 μM taurine was 163.6 ± 3.7, and 1 mM taurine was 203.4 ± 2.4 (*p* < 0.00001 for control vs. 50 μM taurine, and *p* < 0.00001 for 50 μM taurine vs. 1 mM taurine). **(C)** The intensity of PSD95 puncta was significantly decreased in neurites cultured in 50 μM taurine. The intensities of PSD95 puncta in control was 116.2 ± 4.0, in 50 μM taurine was 97.6 ± 3.6, and in 1 mM taurine was 117 ± 2.9 (*p* = 0.002 for control vs. 50 μM taurine, and *p* = 0.0001 for 50 μM taurine vs. 1 mM taurine). **(D,E)** The number of presynaptic synaptophysin and postsynaptic PSD95 puncta did not differ significantly between any treatments. Specifically, the number of synaptophysin puncta was 22.8 ± 2.3 for control, 25.3 ± 2.6 for 50 μM taurine, and 22.3 ± 2.4 for 1 mM taurine (*p* = 0.684). The number of PSD95 puncta was 7.9 ± 1.3 for control, 8.6 ± 1.5 for 50 μM taurine, and 9 ± 1.2 for 1 mM taurine (*p* = 0.852). **(F)** The area of synaptophysin puncta was measured and resulted in a significant increase for 1 mM taurine-treated neurons. The area of synaptophysin puncta for control was 3.8 ± 0.2 μm^2^, for 50 μM taurine was 4.3 ± 0.2 μm^2^, and for 1 mM taurine was 5.8 ± 0.3 μm^2^ (*p* < 0.00001 for control vs. 1 mM taurine, and *p* = 0.00002 for 50 μM taurine vs. 1 mM taurine). **(G)** Taurine treatment at 50 μM decreased the PSD95 puncta area, leading to a significant increase in puncta area of 1 mM taurine-treated neurites compared to 50 μM treatment. The area of PSD95 puncta was 5.3 ± 0.7 μm^2^ for control, 4.4 ± 0.3 μm^2^ for 50 μM taurine, and 6.4 ± 0.5 μm^2^ for 1 mM taurine (*p* = 0.01 for 50 μM taurine vs. 1 mM taurine; *n* = 13, 15, and 22 cropped, neurite images analyzed The area of PSD95 puncta for control was 50 μM taurine, and 1 mM taurine, respectively; the statistical test was one-way ANOVA in all cases). **Indicates a significance level of *p* < 0.01 vs. control, *****p < 0.00001, and ^#^indicates a significance level of *p* < 0.05 vs. 50 μM taurine, ^###^*p* < 0.001, ^####^*p* < 0.0001, and ^#####^*p* < 0.00001.

**Figure 5:**
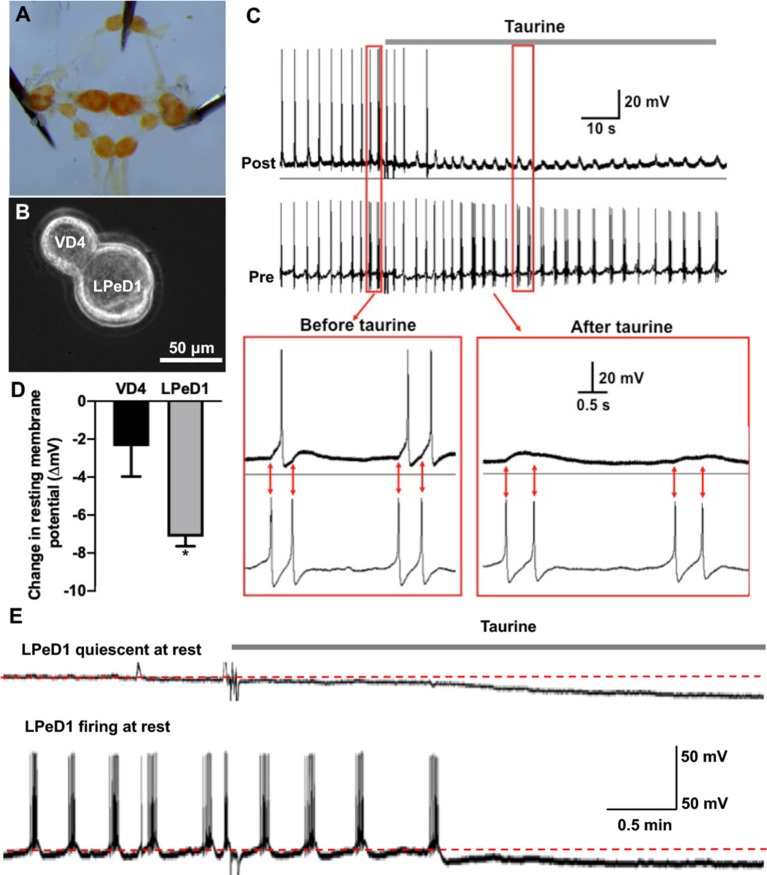
Acute exposure of *Lymnaea* central neurons to taurine alters the excitability of two synapse-forming neurons. **(A)** *L. stagnalis* central ring ganglion was dissected. **(B)** To examine whether acute exposure to a high concentration of taurine affects neuronal excitability and/or synaptic transmission, well defined *L. stagnalis* pre- and postsynaptic VD4-LPeD1 neuronal pairs were cultured and allowed to form synapses (*n* = 4). **(C)** Intracellular recordings revealed that presynaptic action potentials elicited electrical activities in postsynaptic neurons, and these were quieted after exposure to taurine at 2.5 mM. Interestingly, the postsynaptic potentials (PSPs) remained throughout the presence of taurine, indicating that taurine can selectively modulate neural excitability change while allowing the synaptic transmission to occur between two synaptic neurons. **(D)** At rest, taurine caused a significantly larger hyperpolarizing membrane potential change (ΔmV) in LPeD1 neurons (7.14 ± 0.5 mV) than that in VD4 neurons (2.37 ± 1.6 mV; student *t-test*, *p* = 0.049; *n* = 3). Negative direction and Y-axis values indicate a hyperpolarizing action of taurine on the resting membrane potentials of VD4 and LPeD1. **(E)** The hyperpolarization in LPeD1 neurons occurred in either quiescent or actively firing LPeD1 cells. The dotted lines indicate basal membrane potential levels. *Indicates a significance level of *p* < 0.05.

### Immunocytochemistry and Confocal Microscopy

Immunostaining was performed to examine morphological changes in the neuronal cytoskeletal and synaptic proteins of cortical neurons cultured for 3 days and 10 days, respectively. Neurons were fixed with 4% paraformaldehyde and 15% picric acid for 1 h at room temperature. Permeabilization of fixed cultures was performed by incubation for 1 h in incubation media containing 5% goat serum and 0.1% Triton X. Cells were then incubated with primary antibodies rabbit anti-synaptophysin (1:500, Abcam) and mouse anti-PSD95 (1:2,000; NeuroMab) to study the development of synaptic proteins. Primary antibodies were applied at 4°C overnight. Following 3× wash with 1× PBS, secondary antibodies AlexaFluor 488 goat anti-rabbit IgG (1:200; Invitrogen) and AlexaFluor 546 goat anti-mouse IgG (1:200; Invitrogen) were applied for 1 h at room temperature. Mouse anti-β-tubulin (1:500; Invitrogen) was used to study the development of cytoskeletal proteins. Preparations were washed 3× in 1× PBS and mounted with MOWIOL mounting media. A Zeiss confocal microscope (LSM 510 Meta, Zeiss, Germany) was used to take fluorescence images. Image acquisition parameters such as exposure times, gain settings, laser intensity, pinhole size, etc., remained the same between control and treated cultures.

### ImageJ Neurite Tracing and Synaptic Puncta Analysis

ImageJ was used to analyze phase contrast and immunofluorescent images of cortical cells. The ImageJ plugin NeuronJ was employed to measure neurite growth in phase-contrast images of cortical neurons 3 days in culture as previously described (Meijering et al., 2004; Pemberton et al., 2018). NeuronJ was programmed to output total neurite outgrowth (μm) and the number of neurites per phase-contrast image. Therefore, after counting the total number of cell bodies per image, the average neurite length was calculated by dividing the total neurite outgrowth by the total number of cell bodies. Using β-tubulin fluorescently stained day three cortical neurons, ImageJ was used to determine total β-tubulin intensity (IntDen output; the product of area and mean gray value) per image. From the same β-tubulin fluorescently stained neuronal images, primary neurites extending from pyramidal neurons were identified. The thickest part of each neurite was measured to compare the average neurite thickness. Using this method, 30–40 primary neurites were measured for thickness in each treatment.

Cortical neurons were cultured taurine-free or with 50 μM or 1 mM taurine, fluorescently labeled with the presynaptic marker synaptophysin and the postsynaptic marker PSD95 after 10 days in culture, and imaged. Synaptophysin and PSD95 fluorescent intensity were measured in ImageJ as described for β-tubulin above. The ImageJ plugin SynapCountJ was used to measure the number of synapses (colocalization of synaptophysin and PSD95) per area of traced neurite as previously described (Mata et al., 2016). Synaptic puncta parameters were measured with the ImageJ plugin SynQuant (Wang et al., 2020). To look at synaptophysin (presynaptic) and PSD95 (postsynaptic) puncta measurements in entire fields of view (many neuronal networks taken together), uncropped fluorescent images were analyzed by SynQuant, and the number of synaptophysin and PSD95 puncta was outputted. To focus on neurites directly, primary neurites extending from pyramidal neurons were identified. A 50 μm × 20 μm square was placed on the start of primary pyramidal neurites (directly extending from the cell body) and cropped to create a 1,000 μm^2^ area of a zoomed-in neurite. With this method, 13–22 neurites were measured per treatment. These neurite images were analyzed by SynQuant to measure number, intensity, and area of synaptophysin and PSD95 puncta.

### Electrophysiology

Intracellular recording techniques were used to investigate neuronal excitability and synaptic physiology between the paired *Lymnaea* neurons. Glass microelectrodes (1.5 mm internal diameter; World Precision Instruments) were pulled using a vertical pipette puller (Model 700C, David Kopf Instruments). The electrodes were backfilled with a saturated solution of K_2_SO_4_ to yield a tip resistance ranging from 30 to 60 megaohms. Neurons were viewed under an inverted microscope (Axiovert 200 M; Zeiss) and impaled by Narishige micromanipulators (MO-202, Narishige). Electrical signals were amplified with a Neuro data amplifier (Neuron Data Instrument Corp) and recorded with the Axoscope program (Axon Instruments).

Using the *Lymnaea* synapse model in combination with intracellular recording techniques, we asked the following questions: Does taurine affect synapse formation (synaptogenesis), synaptic transmission, and synaptic plasticity between *Lymnaea* neurons? Does taurine exhibit synergistic actions with trophic factors to affect synaptic properties in invertebrate neurons? Does taurine act on the presynaptic or postsynaptic site?

#### Synaptogenesis Experiments

To evaluate the effects of taurine on synapse formation, presynaptic visceral dorsal 4 (VD4) and postsynaptic left pedal dorsal 1 (LPeD1) cells in *L. stagnalis* were paired and cultured in the absence or presence of 1 mM taurine (Sigma-Aldrich) in DM (no trophic factors) or CM (with trophic factors) overnight. Intracellular recordings were made the next day to monitor the development of synapses by recording postsynaptic potentials (PSPs) in the LPeD1 cell following the presynaptic stimulus. Current injection-induced action potentials in the presynaptic VD4 neuron triggered 1:1 PSPs in the postsynaptic LPeD1 neuron, indicating the formation of functional synapses. The current injection was made using a built-in current injector (Dual Channel Intracellular Recording Amplifier IR-283; Cygnus Technology, Delaware Water Gap, PA, USA). Averages of four successive PSPs measured from treated and untreated control groups were compared to determine the effects of taurine on the incidence and strength of synapse formation. The percentage of synapse formation was determined as the number of pairs that exhibited quantifiable transmission of stimuli between cells out of the total number of pairs that were treated.

#### Synaptic Transmission Experiments

To evaluate the effects of taurine on synaptic transmission at established synapses, VD4-LPeD1 neurons were cultured in the absence or presence of taurine (1 mM) in DM or CM overnight. Electrophysiological recordings were collected as described above to determine baseline synapse strength. Average amplitudes of PSPs were measured from control or treatments and compared to determine the effects of taurine, CM, or their combination on the strength of the synaptic transmission. The membrane potential of the LPeD1 neuron was maintained at −100 mV by current injection to enable a comparative evaluation of synapse strength.

#### Synaptic Plasticity (Post-tetanic Potentiation) Experiments

**Figure 6:**
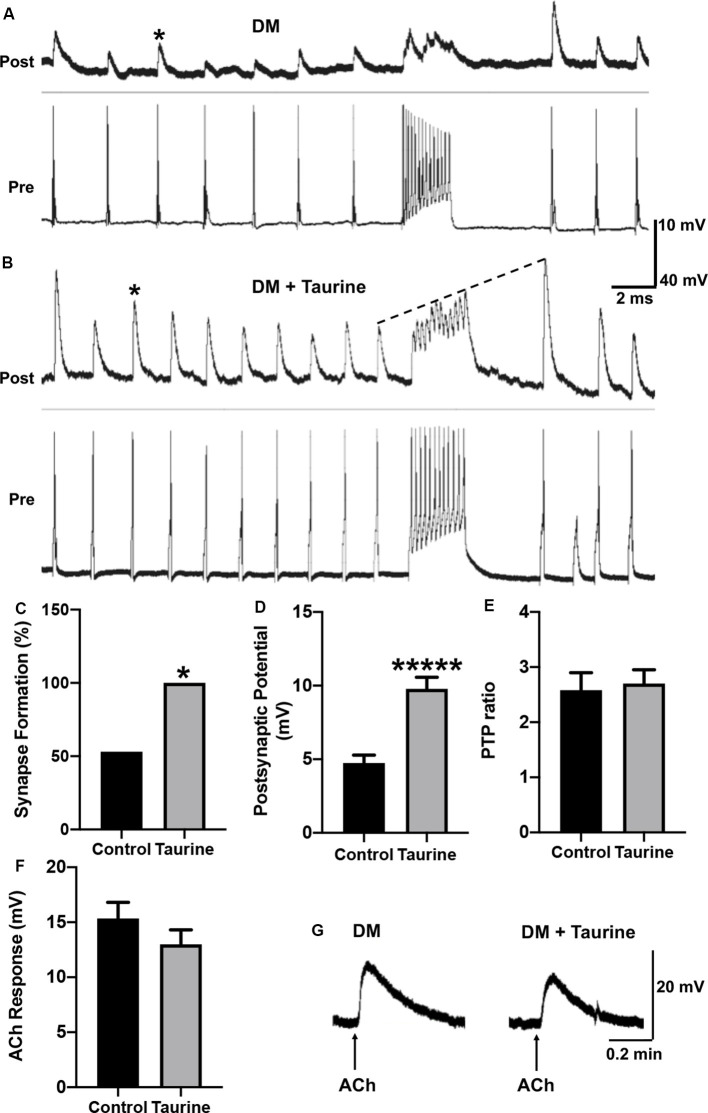
Taurine enhances the development and strength of functional synapses between *L. stagnalis* central neurons but does not affect the postsynaptic response of exogenously applied transmitter. **(A,B)** Representative recordings show the responses of cell pairs cultured without taurine (control) and with 1 mM taurine. Asterisks denote peaks of PSPs. **(C)** Quantification of the incidence of synapse formation (reflected by the percent of cell pairs exhibiting 1:1 ratio of presynaptic action potential: postsynaptic cell PSPs) demonstrated that taurine enhances synaptic incidence. Specifically, 53% of control pairs formed synapses while 100% of 1 mM taurine-treated pairs formed synapses (*n* = 15 for control and *n* = 10 for 1 mM taurine; Fisher’s exact test, *p* = 0.02019). **(D)** Statistical analyses of the efficacy of synaptic transmission (the mean amplitudes of PSPs) showed taurine significantly enhances synaptic transmission strength. The control PSP was 4.74 ± 0.54 mV and 1 mM taurine was 9.78 ± 0.79 mV (*n* = 8 for control and *n* = 10 for 1 mM taurine; student *t*-test, *p* < 0.00001). **(E)** Post-tetanic potentiation (PTP) ratio of postsynaptic potential before and after tetanic stimulation (PSP to pPSP ratio) was not significantly different between control neurons and neurons treated with taurine. The control PTP ratio was 2.58 ± 0.32 and 1 mM taurine was 2.7 ± 0.25 (*n* = 8 for control and *n* = 10 for 1 mM taurine; student *t*-test, *p* = 0.7684). The dotted line in B indicates an example of PTP in which the pPSP after high-frequency stimulation is higher than PSP before high-frequency stimulation. **(F)** To determine if taurine-induced increases in *Lymnaea* synaptogenesis and transmission involves the promotion of expression or function of postsynaptic transmitter receptors, LPeD1 cells were cultured in the absence or presence of taurine overnight. Intracellular recordings were made the next day and ACh (1 μM) was pressure-injected onto the cell bodies of LPeD1 while neurons were held at −100 mV. There was no significant difference between membrane potential responses in taurine-free (15.34 ± 1.46 mV; *n* = 17) and taurine-treated (12.99 ± 1.32 mV; *n* = 10) neurons (student *t-test*, *p* = 0.2393). **(G)** Examples of raw traces of postsynaptic membrane potential response to exogenously applied ACh in a neuron cultured in the absence or presence of taurine (1 mM). *Indicates a significance level of *p* < 0.05, and *****p < 0.00001.

**Figure 7:**
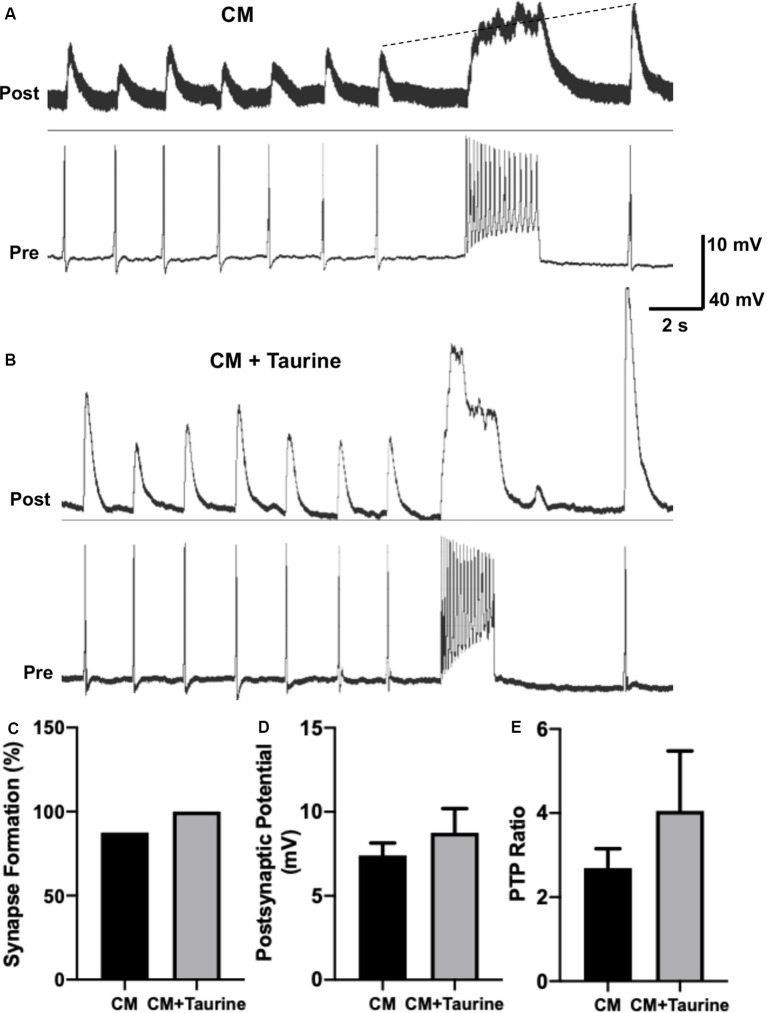
Taurine nonsignificantly increases *L. stagnalis* neuron synapse formation, transmission, and plasticity in medium rich with trophic factors. *L. stagnalis* neurons were paired in a soma-soma configuration and cultured in medium containing *L. stagnalis* derived neurotrophic factors in *Lymnaea* brain conditioned medium (CM) alone or CM plus 1 mM taurine (CM + taurine). Intracellular recordings were made from the cell pairs. **(A,B)** Representative recordings show the responses of *L. stagnalis* cell pairs cultured in CM alone or CM + taurine. **(C)** Incidence of synapse formation was calculated as the percentage of cell pairs forming a 1:1 ratio of presynaptic action potential to postsynaptic PSP; cells cultured in CM + taurine formed strong synapses in all the pairs (100%) examined, while 87.5% of the pairs formed synapses in CM alone (*n* = 5 for CM and *n* = 8 for CM + taurine; Fisher’s exact test, *p* = 1). **(D)** Average peak amplitudes of PSPs of cells cultured in CM + taurine was increased compared to cells cultured in CM alone. PSP in CM cultured cells was 7.40 ± 0.74 mV while PSP in CM + taurine cultured cells was 8.75 ± 1.44 mV (*n* = 7 for CM and *n* = 5 for CM + taurine; student *t*-test, *p* = 0.4107). **(E)** The ratio of postsynaptic potential before and after tetanic stimulation (PTP ratio) was larger in pairs cultured in CM + taurine than those cultured in CM alone, although this was not significant. PTP ratio for CM was 2.69 ± 0.46, and PTP ratio for CM + taurine was 4.05 ± 1.43 (*n* = 7 for CM, and *n* = 5 for CM + taurine; student *t*-test, *p* = 0.4069). The dotted line in **(A)** shows the increase in pPSP after high-frequency stimulation.

VD4-LPeD1 neurons were soma-soma juxtaposed and cultured in the absence (control) or presence of treatment overnight. Induced action potentials in the VD4 neuron triggered 1:1 PSPs in the postsynaptic LPeD1 neuron. Following a tetanic stimulation (a tetanic burst at 10 Hz), the PSP amplitude post-tetanus (pPSP) was substantially potentiated. The increase in the pPSP/PSP ratio is defined as (PTP; as shown in Figures 6, 7), which underlies short-term synaptic plasticity. The pPSP/PSP values were recorded in neurons cultured in the absence (control) or presence of taurine (1 mM) in DM or CM. The tetanic stimulation was generated by injecting a square depolarizing current pulse in a duration of about 2 s into the presynaptic cell to elicit 12–16 action potentials, a well-defined stimulation paradigm for inducing consistent PTP as described in previous studies (Luk et al., 2011).

#### ACh Puffing Experiments

To further elucidate if taurine’s action on synapse development and synaptic transmission involves the regulation of postsynaptic nAChRs, the transmitter ACh (1 μM) was pressure-applied (15 Psi, 100 ms duration) onto LPeD1 neurons to mimic transmitter release through a glass pipette (~2 μm tip in diameter) which was connected to a PV800 Pneumatic Picopump (World Precision Instruments). Intracellular recordings were made on LPeD1 cells (held at −100 mV) to monitor the membrane potential change in response to the puffed ACh. The pipette containing ACh was placed at a distance of about two soma-lengths from LPeD1 to avoid mechanical disturbance.

ACh (A-2661), taurine (T8691), and all other chemicals were purchased from Sigma Aldrich (Saint Louis, MO, USA).

### Statistical Analysis

ImageJ was used to analyze morphological structures of neurites and synapses in cortical neurons with phase-contrast images and immunofluorescent-labeled β-tubulin, synaptophysin, and PSD95. Mini Analysis software (Synaptosoft) was used to measure the amplitudes of PSPs. RStudio was used to run statistical significance tests. Data were statistically analyzed using Fisher’s exact test, student *t*-tests, one-way analysis of variance (ANOVAs), and Tukey’s HSD *Post hoc* tests as appropriate. Values were considered statistically significant at the level of *p* < 0.05. The data are presented as mean ± S.E.M. Each experiment was replicated a minimum of three times; the actual number of replicates for each experiment is described in the text or listed in the corresponding figure legend. All graphs/figures were made using GraphPad Prism 8.4.2 and Adobe Photoshop 2020.

## Results

### Taurine Promotes Neuritic Growth in Rat Cortical Neurons

Taurine has been shown in previous studies to promote neurogenesis and neural progenitor cell (NPC) proliferation in both developing and adult mouse brains (Hernandez-Benitez et al., 2012). However, less is known about the effect of taurine on the development of neural processes such as dendrites and axons. Also, no studies have examined taurine’s effects on neuritic development in brain regions such as the vertebrate cortex. To fill this knowledge gap, we first investigated whether taurine affects neuronal growth of developing rat cortical neurons. To this end, rat cortical cells from day 0 pups were cultured either in the absence or presence of taurine at physiological concentrations of 50 μM and 1 mM for 3 days. Phase-contrast images (Figure 1) were taken on day 3 to evaluate the effect of taurine on cells and the development of neuritic processes. We found that neurons cultured in taurine at concentrations of 50 μM and 1 mM exhibited healthy cell bodies with neuritic processes that formed complex networks. Figure 1A provides representative phase-contrast images of cultures treated without (control) and with taurine at 50 μM and 1 mM. To further define the effects of taurine on neuritic morphogenesis, we conducted neurite tracing and counting analyses using ImageJ neurite tracing methods (Meijering et al., 2004; Imaninezhad et al., 2018; Pemberton et al., 2018). Specifically, we counted and measured the number of neurites and the total neurite outgrowth (the neuritic lengths measured in each image). The total number of cell bodies was also counted and used to calculate the average length of a neurite per cell. Figures 1B–E show quantitative data and statistical analyses. Overall, our ANOVA statistical data revealed that compared to controls, taurine treatment at both concentrations significantly increased the total neurite outgrowth (*p* < 0.001) per image (but not per cell), the total number of cells (*p* < 0.05), and the total number of neurites (*p* < 0.01) in cultures.

To further confirm and define the positive effects of taurine on the morphogenesis observed in our phase-contrast study, we next performed an immunocytochemical study on fluorescently labeled neurites with an antibody against cytoskeletal protein β-tubulin, a microtubule protein component that exists in all neurites and plays essential roles for neuritic formation, growth, and network function. Figure 2A shows representative images of β-tubulin-stained day 3 cortical neurons. These cultures revealed robust expression of the cytoskeletal protein in both control and taurine (50 μM and 1 mM)—treated neurons. Consistent with our phase-contrast images, these fluorescent images revealed extensive neuritic processes (indicated by arrows) and interconnections (asterisks; Figure 2A). Next, we used ImageJ to analyze β-tubulin immunofluorescent images and conducted statistical analyses to determine if taurine treatments affect cytoskeletal expression (measured by the fluorescent intensity) and subtle changes of neurite thickness. Our data showed that taurine treatment at both concentrations significantly increased the thickness of neurites (*p* < 0.001) but did not affect the intensity of β-tubulin protein expression in neurons cultured for 3 days in treatment (Figures 2B,C). Intriguingly, though, neurons cultured in 1 mM taurine demonstrated significantly increased β-tubulin intensity compared to controls after 6 days in culture, suggesting that taurine may increase β-tubulin protein expression over developmental time (data not shown).

Neuritogenesis and cytoskeletal development are essential steps toward the functional development of cell-cell contacts (synapses), synaptic transmission, and plasticity. Next, we asked the question: Does taurine play a role in enhancing the synaptic properties of neurons?

### Taurine Regulates the Expression and Punctualization of Synaptic Machinery Proteins in Cortical Neurons

To directly examine the role of taurine in synapse development, we asked whether taurine affects the development of molecular machinery at the synaptic sites of rat cortical neurons. To test this, neurons treated with taurine at the above-mentioned concentrations were maintained in culture for 10 days to allow the establishment of synaptic networks. Neurons were then fixed and stained with synaptic markers synaptophysin (presynaptic vesicle membrane-associated protein) and postsynaptic density protein 95 (PSD95, postsynaptic membrane-associated scaffolding protein). Figure 3A shows representative images of neurons treated with and without 50 μM or 1 mM taurine. Both synaptophysin and PSD95 exhibited puncta expression and distribution in neurites. To quantify taurine’s effect on these synaptic proteins, we measured synaptic parameters including fluorescent intensity, number of individual puncta, and number of overlapped puncta (synapses) of synaptophysin and PSD95 in the entire image area. Our results revealed that taurine at 1 mM, but not 50 μM, significantly increased the expression of presynaptic synaptophysin protein compared to control (Figure 3B). Taurine at 1 mM also caused an increase in PSD95 expression, but this increase was not statistically significant (Figure 3C). Consistently, our data showed that the number of synaptophysin puncta, but not PSD95 puncta were increased significantly by taurine at 1 mM, but not 50 μM (Figures 3D,E). However, our data did not reveal a significant difference in the number of overlapped puncta of synaptophysin and PSD95 (number of synapses; Figure 3F). Thus, our data analyses indicate that taurine at a higher concentration of 1 mM selectively enhances the expression of presynaptic synaptophysin protein and the number of synaptophysin puncta.

To gain further insight into the effect of taurine on synaptic machinery, we next focused on analyzing neurites (cropped 50 μm × 20 μm section of neurites extending from pyramidal neurons; see “Materials and Methods” section for detailed description) using the SynQuant plugin in ImageJ (Wang et al., 2020). This method allows for the identification of puncta and the detailed quantification of the puncta intensity and area (Figure 4A). Our data showed that although the average number of synaptophysin and PSD95 puncta per neuritic area was not significantly different between taurine treatments and controls (Figures 4D,E), the intensity (Figure 4B) and area (Figure 4F) of synaptophysin puncta were significantly increased by taurine at 1 mM compared to controls. Again, taurine at 50 μM did not affect synaptophysin puncta as compared to controls. Interestingly, taurine at 50 μM decreased the intensity (Figure 4C) and area (Figure 4G) of PSD95 puncta, while 1 mM taurine increased PSD95 puncta intensity and area compared to controls, though not to a significant degree. These data indicate that taurine increased the expression and probably the size (reflected by the area) of individual presynaptic puncta, but not the postsynaptic puncta. Taken together, our data indicated that taurine plays an important role in promoting synaptic machinery development by selectively acting on synaptic proteins to enhance protein expression and puncta development in cortical neurons.

While these data provide the first direct morphological evidence that taurine promotes growth and development of synaptic structures in cortical neurons, direct measurements of neural activity and synaptic properties at the level of single pre-and postsynaptic neurons in vertebrates are not feasible here; we thus opted to use an invertebrate model where this could be done in functionally defined, individual large neurons in the absence of glia and confounding factors including taurine’s analogous transmitters GABA and glycine.

### Taurine Regulates Neural Excitability and Synaptic Activity Between Two *Lymnaea* Neurons That Form Functional Synapses *in vitro*

To accurately monitor the effects of taurine on the functional development of synapses between identified individual neurons, we first determined if taurine indeed has a functional impact on neuronal excitability and transmission in *Lymnaea* neurons whose synapses have been established. To test this, *L. stagnalis* visceral dorsal 4 (VD4, presynaptic, acetylcholine-containing neuron) and left pedal dorsal 1 (LPeD1, postsynaptic) cells were paired in the soma-soma configuration (Figure 5B) and were first allowed to develop proper excitatory cholinergic synapses overnight in CM (contains *Lymnaea* brain secreted neurotrophic factors, but devoid of taurine). The next day, intracellular recordings were made from both neurons. In one experimental paradigm, we tested if taurine could regulate neuronal action potential firing and synaptic transmission between VD4 and LPeD1. In another experimental paradigm, we examined if taurine affects the resting membrane potentials of VD4 and LPeD1 cells. To elicit neuronal activity, we selectively injected depolarizing currents into the presynaptic VD4 neuron to induce action potentials in VD4. Current injection into the postsynaptic LPeD1 neuron brought its membrane potential close to threshold potentials to increase the likelihood of action potential firing in LPeD1 in response to presynaptic excitatory neurotransmitter (ACh in this case). Figure 5C shows that current injection-induced, presynaptic action potentials triggered the firing of action potentials on the postsynaptic cells (see inserts, synchronizing the firing). Next, we added taurine to test if it could affect the firing activity in either or both neurons. Our results showed that upon application of taurine at 2.5 mM, spontaneous firing activities in the postsynaptic LPeD1 cells were quickly prevented in all cells examined (*n* = 4), while firings in the presynaptic VD4 neurons remained active in three out of four cells with one cell eventually stopping firing after taurine application. Interestingly, while the spontaneous firing of the postsynaptic neurons stopped after taurine addition, it still exhibited 1:1 postsynaptic potential (synaptic transmission) in response to presynaptic action potentials for as long as VD4 action potentials remained (see Figure 5C). Furthermore, our data showed that when cells were held at resting membrane potential (no current injection applied in either cell), taurine only caused a small membrane hyperpolarization in VD4 cells and significantly larger membrane hyperpolarization in LPeD1 cells (student *t-test*, *p* < 0.05; Figure 5D), regardless if LPeD1 was quiescent or actively firing at rest (Figure 5E). Overall, these results indicate that taurine can induce an inhibitory or hyperpolarizing change in membrane excitability; also, these results indicate that taurine-induced effects are cell-specific, with there being more pronounced effects on the postsynaptic LPeD1 cell than that of presynaptic VD4 cell. Our data further indicate that taurine’s effects on membrane excitability do not exclude the normal synaptic transmission and plasticity between two synaptic neurons. Together, these studies strongly suggest that taurine does indeed regulate neuronal excitability and synaptic activity in *Lymnaea* neurons.

### Taurine Increases the Incidence of Synaptogenesis and Enhances Synaptic Strength Between Cultured Pre- and Postsynaptic Neurons From Invertebrate *Lymnaea*

We next examined if taurine could functionally regulate the formation of synapses (synaptogenesis), synaptic transmission, and plasticity using *L*. *stagnalis* central neurons. Such information is important for our understanding of taurine’s roles in the development and function of invertebrate nervous systems, an area that remains under-explored. Also, results from *L*. *stagnalis* synapse studies help answer the question of whether the role of taurine is evolutionally conserved between vertebrate and invertebrate nervous systems. With our above and previous evidence that taurine promotes the development of synaptic machinery in mammalian neurons (Shivaraj et al., 2012), we hypothesized that taurine would promote the functional development of synapses between *L. stagnalis* neurons, and its effect may be pre- or postsynaptic site-specific.

To test the above postulate, *Lymnaea* VD4 and LPeD1 cells were paired in the soma-soma configuration (Figure 5B) in the absence or presence of taurine overnight at 1 mM, a concentration that consistently promoted the development of rat cortical neurites and synaptic structures in the above experiments. The next day, intracellular recordings from both cells were made to determine synapse formation and synaptic transmission (representative recordings are shown in Figures 6A,B). Calculating the percentage of cell pairs forming functional synapses demonstrated that taurine significantly increased the incidence of synapse formation between cultured neurons (Figure 6C). The efficacy of synaptic transmission measured by the mean amplitudes of peak PSPs in response to presynaptic current injection-induced action potentials was also significantly increased in neurons cultured in taurine compared to neurons without taurine exposure (Figure 6D). Specifically, our data showed that neurons cultured in the absence of taurine (control) could form synapses in 53% (eight out of 15) of neurons, while all (100%, 10 out of 10) pairs of neurons cultured in 1 mM taurine formed synapses that exhibited robust PSPs (Fisher’s exact test, *p* = 0.02). In cell pairs that formed synapses in the absence of taurine, the mean PSP amplitude was significantly smaller than those detected in taurine-treated neurons (*p* < 0.00001). These data indicate that taurine exerts synaptogenic effects by promoting not only the incidence of synaptogenesis (the increased percent of neuronal pairs that could form synapses) but also the efficacy of synaptic transmission (a single action potential induced a larger postsynaptic response) between individual pre- and postsynaptic neurons.

To further determine the possible role of taurine in synaptic plasticity, we also compared the amplitude of PSP to single presynaptic action potentials before and after a train of high-frequency action potential activity (tetanic stimulation, 10 Hz) in neural pairs cultured with or without taurine. A larger amplitude of action potential-triggered PSP after high-frequency stimulation (pPSP) than PSP before high-frequency stimulation (Figure 6B, indicated by the dotted line) is defined as PTP, a form of short-term synaptic plasticity. Our data showed that taurine did not affect the synaptic plasticity between single neurons (Figure 6E; *p* = 0.7684).

Now the question remains: Does taurine affect synaptic transmission by acting on the presynaptic transmitter release (ACh in this case) or postsynaptic nicotinic ACh receptor (nAChR) expression/response in *Lymnaea* neurons? To help answer this question, we conducted a functional analysis of postsynaptic nAChR response to exogenously applied ACh to mimic transmitter-receptor response *in vitro* (see “Materials and Methods” section). To this end, LPeD1 neurons were cultured either in the absence or presence of taurine at 1 mM overnight to allow for the expression of nAChRs. The next day, neurons were impaled with intracellular sharp electrodes, and ACh (1 μM) was pressure-injected onto LPeD1 cell bodies to monitor the change in membrane potentials (see “Materials and Methods” section and Figures 6F,G). Our results showed that neurons cultured in the presence of taurine did not exhibit a significant membrane potential change in response to ACh application compared to control neurons. Specifically, the mean amplitude of ACh-induced membrane potential in cells cultured in the absence of taurine was 15.33 ± 1.46 mV (*n* = 17) and in the presence of taurine was 12.99 ± 1.32 mV (*n* = 10), which was not statistically different (*p* = 0.2393). These data indicate that taurine-mediated increases in synaptogenesis and synaptic transmission does not involve the promotion of postsynaptic receptor expression/function and most likely involves action on the presynaptic site. However, this experiment cannot rule out the possibility that taurine may also regulate the clustering of postsynaptic receptors to the synaptic sites.

### Taurine Effects on Synaptic Formation, Transmission, and Plasticity Are Comparable to Trophic Factors in Neurons From Invertebrate *Lymnaea*

Because taurine is considered a trophic factor during neuronal development (Sturman, 1993; Chen et al., 1998), we chose to study synaptic properties of *L. stagnalis* neurons that underwent taurine treatment in the presence of trophic factors to deduce if synergistic or additive effects exist. *L. stagnalis* cells were paired in the soma-soma configuration in the presence of medium containing *L. stagnalis* derived neurotrophic factors from *Lymnaea* brain CM, with (CM + taurine; 1 mM) or without taurine overnight. As described above, intercellular recordings (Figures 7A,B) were made from pre- and postsynaptic cells to determine synapse formation, synaptic transmission, and synaptic plasticity. While all (100%, five out of five) pairs of *L. stagnalis* cells formed functional synapses in the presence of CM + taurine, only 87.5% (7 out of 8) paired cells formed functional synapses when cultured in CM alone (Figure 7C; Fisher’s exact test, *p* = 1). The mean PSP amplitude was not significantly different between cell pairs that formed synapses in CM alone or CM + taurine (Figure 7D; student *t*-test, *p* = 0.4107), while the PTP ratio of PSP before and after tetanic stimulation was increased in cell pairs cultured in CM + taurine as compared to cell pairs in CM alone (Figure 7E; student *t*-test, *p* = 0.4069). Synapse formation, synapse transmission, and synaptic plasticity were increased in the presence of taurine, though not to a statistically significant degree, indicating that taurine may act *via* the same or additional pathways as *L. stagnalis* derived neurotrophic factors present in CM.

In summary, our *L. stagnalis* synapse study is supportive of our hypothesis that taurine plays an evolutionarily conserved role vis-à-vis synaptogenesis and synaptic transmission in both invertebrate and vertebrate neurons. Besides, our *Lymnaea* data also indicate that the effects of taurine were comparable to, but not significantly different from trophic factor effects on synaptogenesis, synaptic transmission, and synaptic plasticity between individual *L. stagnalis* neurons.

## Discussion

Early neuronal development relies on many specific events, namely proliferation, migration, differentiation, and synaptogenesis, to achieve proper development of the nervous system. These steps rely on cellular cues such as neurotransmitter release, electrical activity, and binding of molecular ligands (Nguyen et al., 2001; Owens and Kriegstein, 2002; Munno and Syed, 2003; Spitzer, 2006; Batool et al., 2019). As one of the most abundant amino acids in the human body, it is no surprise that taurine has been linked to a variety of physiological functions including neuronal development (Huxtable, 1989; Kilb and Fukuda, 2017). Therefore, we sought to investigate the role of taurine in developing neurons of vertebrate and invertebrate brains. Our data provide the first direct evidence that taurine is a neuroactive substance that specifically impacts neuritic development and synaptic machinery expression and/or assembly, especially the presynaptic machinery. Using an invertebrate model, we demonstrated that acute exposure to taurine regulates neuronal excitability changes between two cultured *Lymnaea* neurons that form mature synapses. With more potent action on the postsynaptic neuron. Chronic (overnight) exposure to taurine at physiological concentrations enhances both the incidence and efficacy of synaptic transmission between two *Lymnaea* neurons, indicating taurine’s *bona fide* synaptogenic function in neurons. Consistent with the cortical neuron synapse data, the chronic action of taurine in *Lymnaea* synapse development is of presynaptic origin. Together, these data demonstrate that taurine’s morphogenic and synaptogenic functions are conserved in both invertebrate and vertebrate species, and taurine’s effects are cell- and synaptic site-specific.

Taurine has been previously shown to promote neurogenesis and NPC proliferation in both developing and adult mouse brains. However, less is known about the effect of taurine on the development of neural processes. In embryonic day 18 (E18) rats, the addition of taurine on day 2 in the culture at 20 μM and 100 μM increased both the neurite length and the number of neurites in day 3 primary hippocampal cells, but not to a significant degree (Shivaraj et al., 2012). In our study, we added taurine at physiological concentrations of 50 μM and 1 mM on day 0 in culture, used postnatal rats (P0), and examined the effects on another brain region, cortical neurons *in vitro* after 3 days in culture. We found a significant enhancement in overall neuritic growth and the number of neurites. The difference in the observed taurine effects on neurites may be attributed to the following factors: difference in brain regions, age of rats during dissection, or taurine treatment protocol (day 0 vs. day 2 exposure). It is interesting that our data also revealed a significant increase in the thickness of primary neurites that were labeled by fluorescent staining of β-tubulin, which has not been reported previously. This increase in the thickness of primary neurites will lead to an increase in neuronal surface area for housing ionic channels/receptors and an increase in the efficiency of neural conductivity and synaptic transmission. Furthermore, our study found that taurine increased the number of cells in culture. This finding is consistent with a previous study showing that taurine treatment caused a significant increase in the number of cells and the length of neurites in primary cultured cochlear spiral ganglion cells (Rak et al., 2014). The increase in spiral ganglion cells was attributed to taurine-mediated neurotrophic effects of higher rates of cell survival of both neurons and glial cells (Rak et al., 2014). In our study, the overall number and length of neurites were increased by taurine, yet the length of neurites per cell was unaffected, further implying that the main function of taurine is enhancing neuronal survival in dissociated cell cultures. While these results suggest a neurotrophic role for taurine in cell survival, it is not known if the increase in cell density we observed is also due to other factors such as a higher rate of adhesion or glial cell proliferation. However, a study by Shivaraj et al. (2012) found that taurine did not affect the number of differentiated glial cells in their hippocampal culture using a specific fluorescent marker of glial fibrillary acidic protein. Nevertheless, it would be interesting to study these possibilities in our future cortical cell culture experiments.

Our data are in fact in agreement with the study by Shivaraj et al. (2012) showing that 100 μM taurine increased synapsin 1, a presynaptic marker, and PSD95 expression in primary mouse hippocampal neurons. Unfortunately, their study only provided evidence of protein level increases using western blotting methods. It, therefore, remains unknown whether taurine affects the level of clustering of synaptic proteins at the single neuron-, neurite-, or network-levels. We took advantage of well-established methods such as SynQuant (Wang et al., 2020) and SynapCountJ (Mata et al., 2016) to analyze different synaptic parameters in entire images (networks) and primary neurites. We found that taurine not only regulated synaptic protein expression in networks but also their intensity in synaptic puncta along neurites. Intriguingly, taurine was more effective at promoting the expression of presynaptic synaptophysin protein than the postsynaptic PSD95 protein. More intriguingly, our exogenous transmitter ACh puff experiments strongly implicate a presynaptic working mechanism in *Lymnaea* synapse development. Our findings are thus consistent with a previous study revealing that taurine selectively acts on the presynaptic NMDA receptors (*via* glycine-binding sites) to potentiate NMDR-induced facilitation of axon excitability and field EPSP in rat hippocampal slices (Suarez and Solis, 2006). It is important to note here that synaptophysin is a ubiquitous, presynaptic marker of all types of synapses, while PSD95 is restricted mainly to glutamatergic synapses. Therefore, a possibility exists that taurine’s presynaptic effects are not restricted solely to a single type of synapse, as it may affect both inhibitory and excitatory. Together, these data indicate that taurine has neurotrophic and synaptogenic effects on developing vertebrate neurons, and taurine effects are more selective on the presynaptic sites.

Our data also revealed that taurine’s effects are more robust and consistent at a higher concentration of 1 mM. It is important to note that concentrations of taurine have been revealed to vary widely between different brain regions and even between pre- and postnatal animals (Palkovits et al., 1986; Miller et al., 2000). Therefore, the differences in the efficacy of taurine at various physiological concentrations seen in the data presented here and previous studies, particularly the E18 hippocampal culture by Shivaraj et al. (2012), is not entirely surprising. While our experiments focused on the effects of physiological levels of taurine on neurons, a recent study demonstrated that chronic exposure to extremely high concentrations of taurine (50 mM) induced cell apoptosis (Serdar et al., 2019), indicating that physiological levels and length of exposure are important factors in taurine’s effects during early neural development. Also, the concentration effects of taurine have been found in taurine’s actions on the regulation of neuronal excitability. For example, taurine has been reported to induce dose-dependent effects on immature hippocampal neurons in both excitatory and inhibitory directions (Chen et al., 2014; Winkler et al., 2019); it enhances neuronal excitability in the immature neocortex (Sava et al., 2014), while it exhibits a dominant, inhibitory effect on neuronal activity in the mature CNS (Kilb and Fukuda, 2017). Also, taurine at mM concentrations inhibits neuronal activity during anoxia in turtle central neurons *via* both glycine and GABA_A_ receptors (Miles et al., 2018). Consistently, our data indicate that taurine at mM concentrations could also negatively regulate neuronal firing activity in *Lymnaea* neurons (Figure 5C) in a cell-specific manner.

In *Lymnaea*, a previous study focusing on taurine’s effect on electrically regulated ionic currents revealed that taurine at mM concentrations decreased Na^+^ currents (hence may reduce firing), yet at low concentrations (100 nM to 100 μM) increased Na^+^ currents (Vislobokov et al., 1991). It is not known if the concentration-dependent effects of taurine on *L. stagnalis* neurons contributed to our observed synapse formation and neuronal activity regulation. For example, the increase of Na^+^ current by lower concentrations of taurine may increase cell excitability and subsequently cause intracellular calcium transients, which is the causal factor of neurotrophic factor-induced synaptogenesis between *Lymnaea* neurons (Xu et al., 2009), while high concentrations of taurine may inhibit Na^+^ currents and reduce neural excitability as seen in our acute exposure experiments (Figure 5C). It is, however, important to note that taurine immediately and completely abolished action potential firing in the postsynaptic but not presynaptic neurons (Figure 5C), suggesting that inhibition of sodium currents alone may not be sufficient to explain the selective action of taurine on the postsynaptic neurons. It is well known that taurine is an agonist of GABA_A_ and glycine receptors, activation of which can counterbalance the neuronal excitability (Herbison and Moenter, 2011; Komm et al., 2014; Kilb and Fukuda, 2017; Ochoa-de la Paz et al., 2019). Thus, taurine may reduce cell excitability potentially *via* GABA_A_ receptors that are expressed in *L*. *stagnalis* central neurons (Harvey et al., 1991; Darlison et al., 1993). Despite the unknown mechanism, our studies, together with others, clearly indicate that taurine acts as an active neuromodulator that is involved in regulating neuronal membrane excitability. The selective inhibition of postsynaptic excitability leads to a less likelihood of synchronized firing of action potentials between pre- and postsynaptic neurons that form excitatory synapses (such as the cholinergic synapses in our study, Figure 5C). More importantly, our data indicate that the hyperpolarization of postsynaptic neurons did not exclude the ability for presynaptic neurons to release transmitter and communicate with the postsynaptic neurons. These reveal novel insights into the role of taurine as a neuromodulator and neuroprotector to aid the interplay between membrane excitability and synaptic transmission, achieving optimal homeostasis of network activity in the nervous system.

Our data show that there is a conserved role of taurine between vertebrates and invertebrates to increase synapse development. Specifically, our data, for the first time, revealed the positive effect of taurine on the percentage of neurons that could form functional synapses and the robustness of synaptic transmission between individual partner neurons. While taurine’s effect on the formation of functional synapses in invertebrate neurons is novel, increases in synaptic transmission have been reported in rat hippocampal slices after treatment with taurine (Galarreta et al., 1996). The effect of taurine on synapses is conserved between vertebrates and invertebrates, further providing evidence that *L. stagnalis* is a good model for studying the roles and cellular mechanisms of taurine on synapses that prove challenging in the mammalian brain.

Taurine has previously been linked to synaptic plasticity, particularly long-term potentiation (LTP), with conflicting results. The addition of taurine mitigated synaptic plasticity impairment in one study (Yu et al., 2007) but failed to alter late-LTP induction in another (Suarez et al., 2016). As a compound with structural similarities to GABA and a partial agonist of GABA_A_ receptors, taurine begs the question of whether it can act on GABA receptors to induce synaptic LTP, as LTP is a previously established characteristic of GABAergic synapses (Gaiarsa et al., 2002; Ochoa-de la Paz et al., 2019). Interestingly, one study reported the ability of taurine to induce LTP in rat hippocampal slices, and the action was independent of GABA_A_ and glutamate receptor activation but relied on the presence of calcium (del Olmo N. et al., 2000; del Olmo N. D. et al., 2000). While taurine’s effects have been linked to LTP, we sought to determine if taurine affects short-term PTP in *L. stagnalis* central neurons. The addition of taurine alone did not alter synaptic plasticity PTP in *L. stagnalis* neurons. However, when taurine was added in combination with trophic factor-rich *Lymnaea* brain CM, synaptic plasticity increased more than *Lymnaea* trophic factors alone, although it did not reach a significant level. These findings might be the result of taurine’s ability to reduce neuronal excitability in concentration-dependent manners as mentioned above. Nevertheless, it is important to note that although synaptic plasticity did not increase significantly between the two individual central neurons we recorded from, this does not mean that synapses among many neurons together cannot exhibit synaptic plasticity after addition of taurine. We found that synaptic puncta and synaptic strength were increased after the addition of taurine, and a possibility still exists that the effect of many neurons within a network increasing synaptic abilities can exhibit a combined effect of synaptic plasticity within the network.

Taken together, our results demonstrate taurine as a stimulator of neuritogenesis and a regulator of synapses in vertebrates and invertebrates, and these effects are cell-specific. The discovery of the conserved developmental and synaptogenic actions of taurine in the *L. stagnalis* nervous system will open research avenues exploring the cellular, molecular, and synaptic mechanisms of taurine’s actions using *L. stagnalis* and other invertebrate models. Particularly, these models accelerate our ability to study molecular mechanisms underlying taurine effects on growth and synapse development between single neurons *in vitro* in the absence of confounding factors including glia and taurine’s analogous transmitters GABA and glycine. Such knowledge is fundamental for future efforts in targeting taurine as a therapeutic agent.

## Data Availability Statement

The raw data supporting the conclusions of this article will be made available by the authors, without undue reservation, to any qualified researcher.

## Ethics Statement

The animal study was reviewed and approved by Institutional Animal Care and Use Policy at the University of Calgary and the Institutional Animal Care and Use Policy at Saint Louis University.

## Author Contributions

BM, FX, and NS designed the study. BM, WZ, and FX performed all experiments. BM and FX performed data analysis, created the figures and wrote the manuscript. BM, WZ, NS, and FX revised and edited the manuscript.

## Conflict of Interest

The authors declare that the research was conducted in the absence of any commercial or financial relationships that could be construed as a potential conflict of interest.
